# Phenylalanine-Assisted Conductivity Enhancement in PEDOT:PSS Films

**DOI:** 10.1021/acsomega.2c07501

**Published:** 2023-02-15

**Authors:** Div Chamria, Christopher Alpha, Ramesh Y. Adhikari

**Affiliations:** † Department of Physics & Astronomy, Colgate University, 13 Oak Drive, Hamilton, New York 13346, United States; ‡ Cornell NanoScale Science and Technology Facility, 250 Duffield Hall, Ithaca, New York 14853, United States

## Abstract

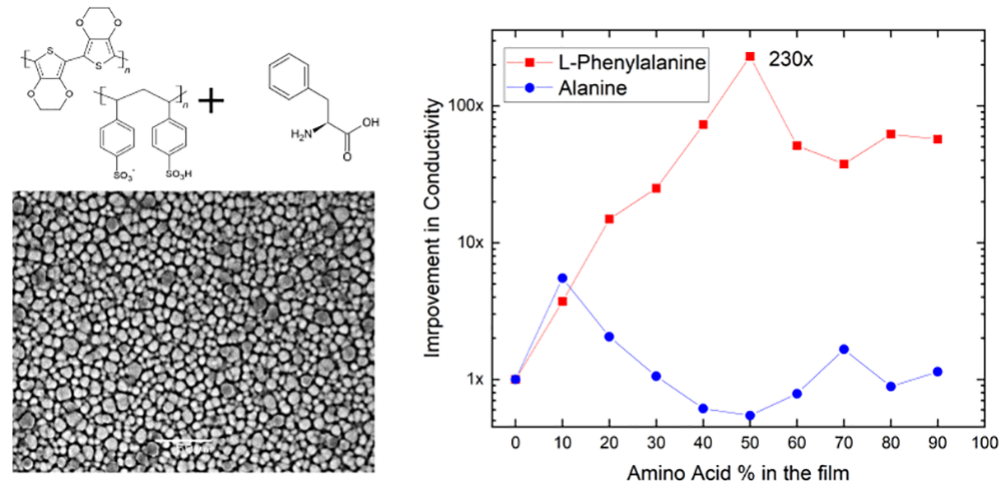

Biological materials such as amino acids are attractive due to their smaller environmental footprint, ease of functionalization, and potential for creating biocompatible surfaces for devices. Here, we report the facile assembly and characterization of highly conductive films of composites of phenylalanine, one of the essential amino acids, and PEDOT:PSS, a commonly used conducting polymer. We have observed that introducing aromatic amino acid phenylalanine into PEDOT:PSS to form composite films can improve the conductivity of the films by up to a factor of 230 compared to the conductivity of pristine PEDOT:PSS film. In addition, the conductivity of the composite films can be tuned by varying the amount of phenylalanine in PEDOT:PSS. Using DC and AC measurement techniques, we have determined that the conduction in the highly conductive composite films thus created is due to improvement in the electron transport efficiency compared to the charge transport in pure PEDOT:PSS films. Using SEM and AFM, we demonstrate that this could be due to the phase separation of PSS chains from PEDOT:PSS globules which can create efficient charge transport pathways. Fabricating composites of bioderived amino acids with conducting polymers using facile techniques such as the one we report here opens up opportunities for the development of low-cost biocompatible and biodegradable electronic materials with desired electronic properties.

## 1 Introduction

Intrinsically conducting polymer complex poly(3,4-ethylenedioxythiophene) doped with poly(styrenesulfonate) (PEDOT:PSS) is widely used in various electronic applications such as active material for organic electrochemical transistors (OECT),^1^ electrodes for supercapacitors,^2^ and photovoltaic cells^3^ owing to their properties such as chemical stability, ease of processing, high transparency as thin films, and commercial availability. In addition to these properties, due to the flexibility of PEDOT:PSS films being mixed electron and ion conductors, they are also currently being studied as sensing surfaces and electrodes for bioelectronic devices.^4−9^

Despite its versatility, the widespread application of PEDOT:PSS in electronic devices is limited due to two major challenges. The conductivity of PEDOT:PSS is lower than that of other commercially available conductors such as indium tin oxide (ITO) and metals.^10,11^ In addition, the use of PEDOT:PSS specifically in bioelectronic devices is also limited by its biocompatibility.^7,12^ To address the first challenge, various research has been carried out to improve the conductivity of PEDOT:PSS.^13−17^ PEDOT:PSS is composed of PEDOT units which are small aggregates with molecular weights of about 1000 g/mol, and PSS units are large with molecular weights of about 400 000 g/mol.^14^ In a PEDOT:PSS film, hydrophobic conducting PEDOT oligomers aggregate along the hydrophilic nonconducting PSS chains that coil to form pancake-like granular structures within which charge transport is promoted by π-stacking of PEDOT units while the charge carriers need to overcome the potential associated with the charge transport across the grains.^14,18−20^ This transport can be enhanced by using various physical and chemical methods that alter the chemical properties or physical morphology of the PEDOT:PSS films.^13,21^ Some examples of such physical methods are thermal treatment,^22^ which increases the grain sizes, and UV radiation,^13^ which linearizes the PSS coil and hence reduces charge trapping. On the other hand, the conductivity of PEDOT:PSS can also be improved by chemically treating PEDOT:PSS with a polar organic compound such as dimethyl sulfoxide (DMSO) or its derivative, ethylene glycol (EG), strong acids, ionic liquids, surfactants, and salts.^10,13,14,19,23,24^ The improvement in conductivity of PEDOT:PSS with these chemical treatments has been attributed to mechanisms such as charge screening, which lowers the Coulombic interaction between PEDOT and PSS, and phase separation between PEDOT and PSS, which removes excess PSS along the charge transport pathways and hence improves charge transport along the PEDOT:PSS film.

To address the issue of biocompatibility, various work has been done by introducing biological materials into PEDOT:PSS or the PEDOT matrix itself.^12,25−27^ Conducting polymers can either be functionalized with biological materials or these biological materials can be introduced as dopants into the conducting polymer matrix. Various bioderived materials such as dextran sulfate^28^ and xanthan gum^29^ have been used as PEDOT dopants, replacing PSS, which results in the formation of conductive biocompatible films. Other bioderived molecules such as pectin,^30^ sulfated cellulose,^31^ and DNA^32^ have also been used as PEDOT dopants. However, these additives either retain or only slightly increase the conductivity of the films thus created. The conductivity of PEDOT:PSS films can also be significantly improved by introducing a bioderived molecule such as DMSO^33^ or sorbitol^34^ which is acquired by processing biological systems.

Keeping in mind the cost-effectiveness of both materials and processes as well as the biological origin, here we report a significant increase in the conductivity of PEDOT:PSS films by doping them with l-phenylalanine (Phe), one of the essential amino acids and building blocks of proteins. Phe self-assembles in water which can be drop cast on a substrate to form a film of mesoscale fibrils and shows no electronic conductance on its own. When the Phe solution in water is mixed with a PEDOT:PSS solution, we have observed that the resulting composite film can have conductivity values of up to two orders of magnitude higher compared to the conductivity of a pristine PEDOT:PSS film. When we repeated the same procedure with alanine (Ala), which shares the molecular structure of Phe but without the aromatic residue, we did not observe such an improvement in conductivity, indicating that the aromatic group in Phe plays a role in the conductivity improvement.

## 2 Results and Discussion

**Figure 1:**
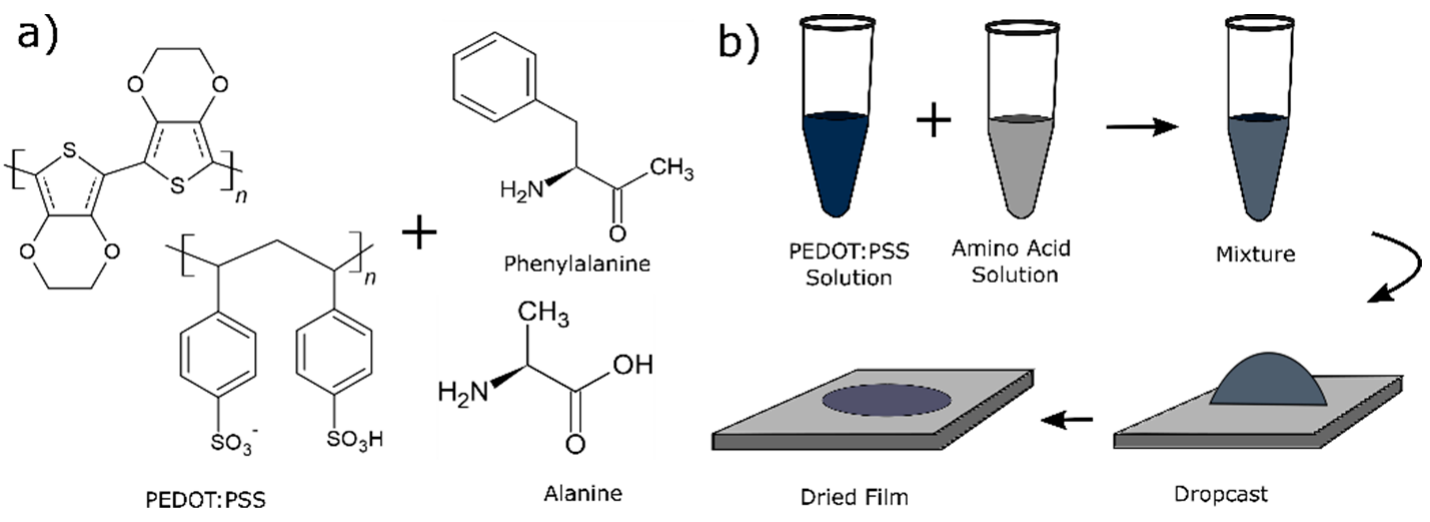
(a) Molecular structures of PEDOT:PSS, Phe, and Ala used in this study. (b) Schematic of the preparation of PEDOT:PSS-Phe (PPP) and PEDOT:PSS-Ala (PPA) films.

We prepared the PEDOT:PSS-Phe (PPP) films by drop-casting solutions that contained a mixture of varying volumetric ratios of PEDOT:PSS and Phe solution (Figure 1). A similar procedure was followed for the PEDOT:PSS-Ala (PPA) films. The details of the preparation process are presented in the Materials and Methods section. We prepared 10 different solutions with the contents ranging from 100 vol % PEDOT:PSS to 0 vol % PEDOT:PSS with the complementary portion being that of Phe or Ala. The drop-casted solution was then left to dry at ambient room temperature and humidity, which resulted in the formation of thin circular films with different levels of coloration due to the difference in the content of PEDOT:PSS (Figure S1).

### 2.1 DC Measurements

**Figure 2:**
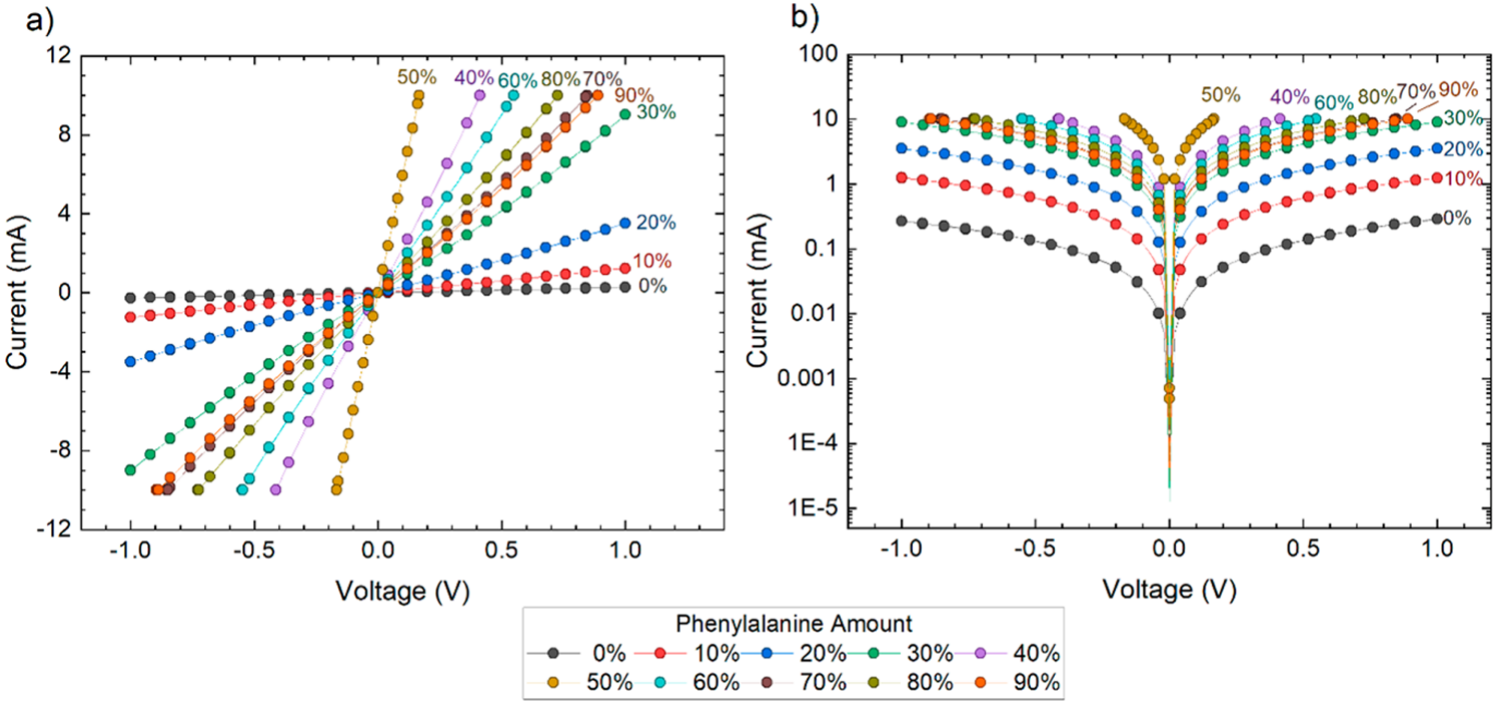
(a) Current–voltage (*I*–*V*) responses of films composed of various volumetric mixtures of PEDOT:PSS and Phe. (b) *I*–*V* response on a semilog graph.

We carried out current–voltage (*I*–*V*) measurements of the films by sweeping voltage across the interdigitated electrodes and then recording the resulting current. The *I*–*V* curves thus acquired were mostly linear and nonhysteretic, indicating the Ohmic behavior of the films (Figure 2a). We observed that the conductance of the film increased even when just a 10 vol % Phe solution was introduced to the film. The current responses were even higher for PPP films with higher concentrations of Phe. In fact, the current response of the films with higher concentrations of Phe was orders of magnitude higher than for pristine PEDOT:PSS films or the films with lower concentrations of Phe even for small applied voltages (Figure 2b). Once the Phe solution used to prepare PPP films exceeded 50 vol %, the conductance started to decrease but still stayed much higher compared to that of pristine PEDOT:PSS films.

**Figure 3:**
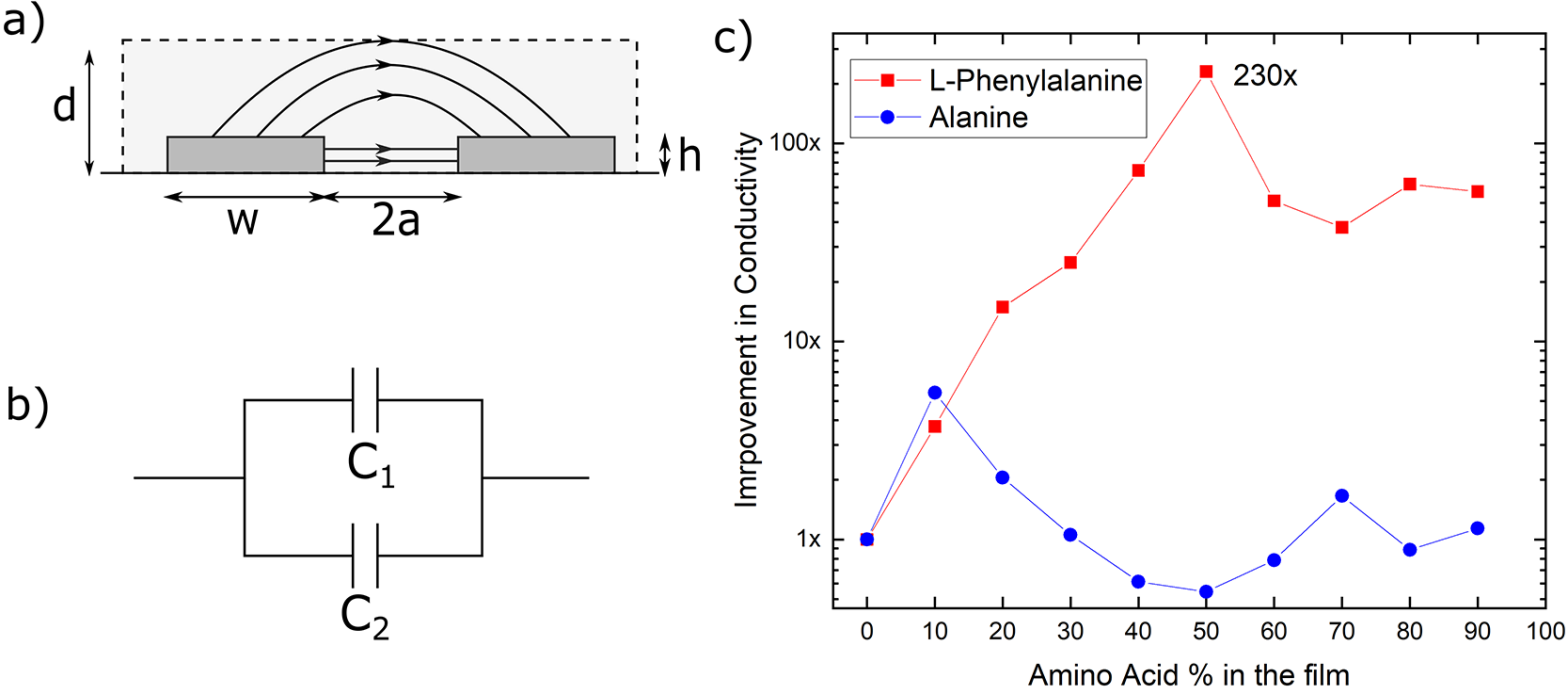
(a) Electric field lines between electrodes when a potential difference is applied. (b) Equivalent circuit model for a pair of electrodes which involves two capacitors. (c) Improvement in conductivity of PEDOT:PSS-amino acid composite film with an increasing percentage of Phe (red) and Ala (blue). 0% indicates films with no amino acid, i.e., with 100% PEDOT:PSS.

In order to measure the conductivity from the conductance values, it is essential to know the geometry of the system involving the sample film and the electrodes as well as a proper model that applies to such geometry. The films we developed had a thickness in the micrometer range, while the interdigitated electrodes we used had an electrode height in the hundreds of nanometers range. When a potential difference is applied across a pair of electrodes, an electric field is generated between those electrodes (Figure 3a). The electric field lines in the space between the electrodes are parallel. However, the electric field lines between the horizontal face of the electrodes are elliptical. Charge transport occurs through the field along these field lines. In all of our cases, the thickness of the films was smaller than the electrode width of 10 μm. Therefore, not all of the elliptical electric fields emanating from the horizontal side of the electrode contribute to charge transport. In such a situation, effective capacitance due to the elliptical field associated with the film thickness is given as^35^
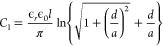
1where ϵ_*r*_ is the permittivity of the film in which the electrodes are embedded, ϵ_0_ is the permittivity of free space, *d* is the thickness of the film, and 2*a* is the gap between the pair of electrodes. On the other hand, the capacitance due to the horizontal electric field between two vertical sides of the electrode is
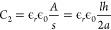
2where *l* is the length of the electrodes and *h* is the height of the electrodes. Therefore, the equivalent capacitance (C*) associated with this system which involves a pair of electrodes would effectively be the capacitance of capacitors in parallel (Figure 3b) and can be written as
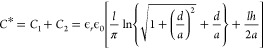
3For interdigitated electrodes with *N* number of electrode fingers, the total number of the gap between the electrodes is *N* – 1 and hence the total capacitance (*C*) of the entire set of the interdigitated electrodes would be
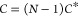
4This capacitance of the system is related to its cell constant (κ) as well as the resistance (*R*) of the embedding film as^36^
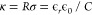
5Therefore, the conductivity of the film (σ) deposited on the electrodes would be
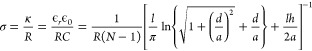
6which we used to calculate the conductivity (Table S1). The conductivity value thus calculated was about 230 times higher for PPP films with 50 vol % Phe solution compared to that of the pristine PEDOT:PSS film (Figure 3c). The conductivity values of the PPP films rose in a roughly logarithmic fashion as seen in the semilog graph (Figure 3c) as the concentration of Phe was increased. This continued until the concentration of the Phe solution reached 50 vol %. Once the concentration of Phe was increased further, the conductivity decreased and then leveled off. Nevertheless, the conductivity of the PPP film made from 90 vol % Phe solution was still about 60 times higher than for a pristine PEDOT:PSS film.

We repeated these measurements on PEDOT:PSS-Ala (PPA) films created by mixing various volumetric ratios of PEDOT:PSS solution with Ala solution. Ala is analogous to Phe, with the only difference in its molecular structure being the lack of aromatic residue in Ala (Figure. 1a). Resulting PPA films also demonstrated linear current responses to sweeping voltages (Figure S2). However, the conductivity of the PPA films (Figure 3c) did not appear to change significantly with changing Ala concentration in the films. The dramatic improvement in the conductivity of PPP films with increasing concentrations of Phe which possesses an aromatic residue, compared to no such improvement in the PPA films with aromatic-residue-deficient Ala, suggests that the aromatic residues in Phe are in some way responsible for promoting efficient charge transport through the PPP system.

### 2.2 Electrochemical Impedance Spectroscopy (EIS)

**Figure 4:**
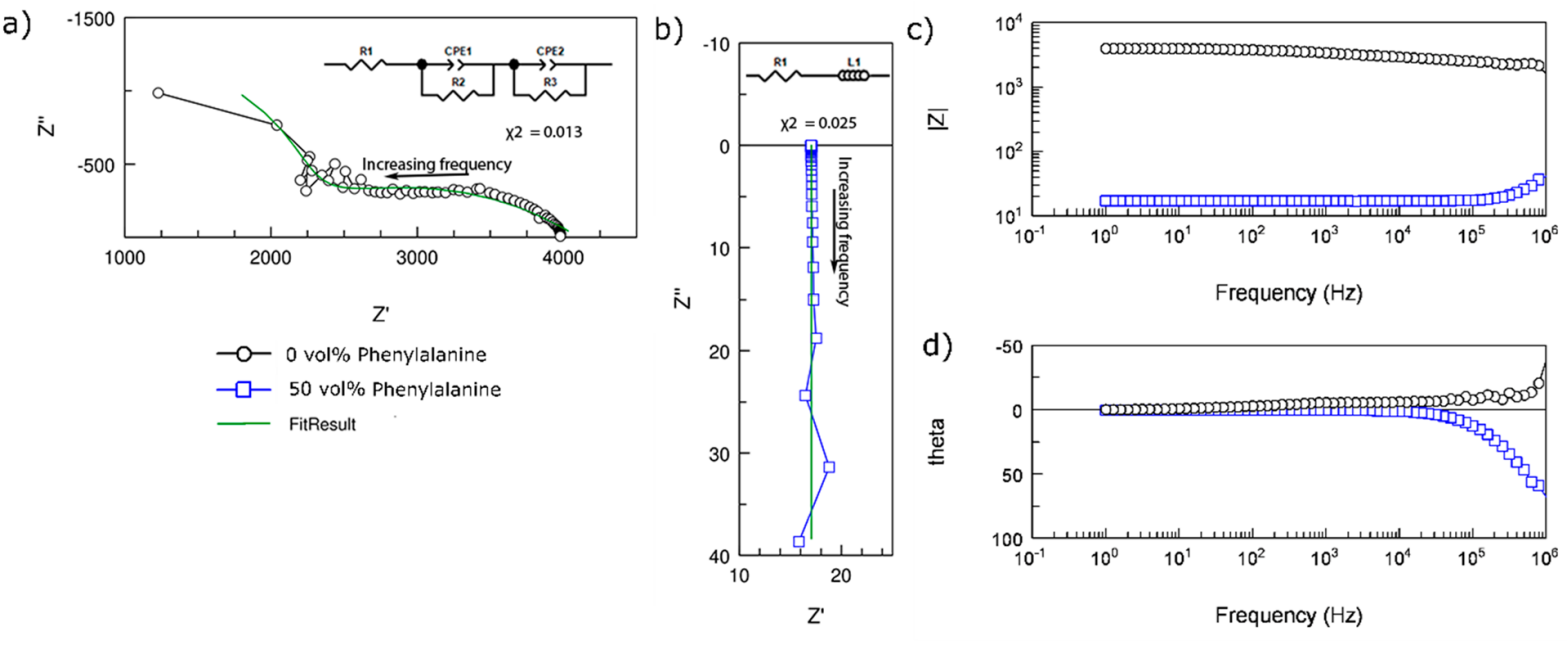
Electrochemical impedance spectroscopy (EIS) measurements: Nyquist plot of (a) a PEDOT:PSS film with the equivalent circuit to fit the data as the inset and (b) a film with a 50% volumetric mixture of PEDOT:PSS and Phe in PPP films with the equivalent circuit to fit the data as the inset. Arrows indicate the direction from low frequency to high frequency regime. Bode plot with (c) the magnitude of impedance and (d) the phase of impedance as functions of frequency.

To further explore the charge conduction mechanisms in these films, we performed electrochemical impedance spectroscopy (EIS) measurements which involve applying time-varying voltage (*V*(*t*)) with a small amplitude across the sample while recording the current response (*I*(*t*)). This can then be used to calculate the impedance of the system which comprises real and imaginary components as *Z* = *V*(*t*)/*I*(*t*) = *Z*′ + i*Z*″. The resulting Nyquist plot of the pristine PEDOT:PSS film demonstrated behavior that can be thought of as a combination of two semicircles with different time constants (Figure 4a). We fit this data with an equivalent circuit comprising two modified Randles circuits in series that include a constant phase element (CPE) in addition to the resistor component (Figure 4a, inset), which resulted in a χ^2^ value of 0.013. Based on the fit values of CPE and the resistors, we found that the time constant (τ = *RC*) for the semicircle toward the higher-frequency regime is 2 × 10^–11^ s and the time constant for the semicircle toward the lower-frequency regime is about 7 × 10^–2^ s, suggesting that there are two different charge transport processes in the film with significantly varying time scales. When Phe was introduced into PEDOT:PSS and then the concentration increased, the two-timescale process evolved into a one-timescale process as the impedance decreased (Figure S3a), with the time constant in the range of 10^–7^ s for 20 vol % Phe in the PPP film. When the Phe content reached 30 vol %, the Nyquist plot collapses into a point on the real impedance axis, suggesting that the capacitive component in charge transport was eliminated and the film became purely resistive.

In the films with an even higher concentration of Phe in the PPP films, the Nyquist plot demonstrates the conduction mechanism in which the resistance is coupled with an inductor instead of a capacitor. This is indicated by the Nyquist plot with the positive imaginary impedance which was prevalent in all of the PPP films with a Phe concentration of 40 vol % or higher (Figure S3b). For instance, the Nyquist plot of the film with 50 vol % PEDOT:PSS, the PPP film with the highest conductivity, demonstrated a high-frequency inductive tail that approached the real axis of the Nyquist plot at lower frequencies (Figure 4b). This was fitted with an R–L circuit and resulted in a χ^2^ value of 0.025. The time constant (τ = *L*/*R*) associated with this transport process was around 10^–7^ s as well. The values of resistance of the PPP films from the EIS measurement, found by fitting with the equivalent circuits, were similar to the values from DC measurements (Table S1), demonstrating the complementary nature of these two charge transport probing mechanisms.

Another method for viewing EIS measurements is through Bode plots where the magnitude of the impedance  and the phase angle of the impedance (θ = tan^–1^ (*Z*″/*Z*′)) are plotted as a function of the frequency. It is clear that the impedance of the PEDOT:PSS film with 50% Phe has an impedance that is orders of magnitude smaller than that of the pristine PEDOT:PSS film for the entire range of frequencies measured (Figure 4c). The magnitudes of the impedance follow the same trend (Figure S4) as observed from DC measurements. Furthermore, the influence of the capacitive behavior for the pure PEDOT:PSS sample is apparent from the negative phase angle toward the higher-frequency end (Figure 4d, Figure S4c). As Phe is introduced into the PPP films, the high-frequency negative phase diminishes, signifying the reduction of the capacitive effect during charge transport. On the other hand, the influence of the inductive behavior in the PPP films with a higher concentration of Phe is apparent due to the positive phase angle toward the higher-frequency regime (Figure 4d, Figure S4d). It is also to be noted that as the concentration of Phe in the PPP films is increased, the positive phase angle associated with the impedance is maximized for PPP films prepared from 50 vol % Phe.

These EIS measurements suggest that Phe in the PPP films enhances charge transport efficiency in the system. Elimination of the capacitive effect by increasing the concentration of Phe in the PPP films with a lower vol % of Phe suggests that the presence of Phe inhibits charge aggregation at the interfaces responsible for creating the capacitive effect. Eventually, no capacitive phenomenon is recorded, suggesting that charge aggregation at the interfaces, such as the interface of the grain boundary or charge trap sites, is completely eliminated. The evolution of two semicircular Nyquist plots into one semicircle suggests that the two-timescale charge transport process is reduced to a one-timescale process with increasing concentration of Phe in the PPP films. With diminishing capacitive processes, the films eventually exhibit purely resistive behavior with low impedance. We have observed that the PPP film with 30 vol % Phe demonstrates purely resistive behavior (i.e., θ ≈ 0°) for the frequencies below the range of 100 kHz (Figure S4c), suggesting highly efficient resistive transport in the film. When the concentration of Phe is further increased, we observe high-frequency inductive tails (Figure 4b, Figure S3b) in the frequency range of 10 kHz or higher (Figure 4d, Figure S4d) but resistive behavior at lower frequencies. High-frequency inductive tails are usually attributed to the inductance of wires and electrodes in the measurement system, which could also partially be the case here.^37^ However, we have observed that the magnitude of the phase associated with the inductance (i.e., θ > 0) for PPP films depends on the concentration of Phe in those films. The highest-conductivity PPP film with 50 vol % PEDOT:PSS shows the highest degree of inductive behavior compared to other PPP films. This suggests that the high-frequency inductive tails in PPP films are potentially due to highly efficient charge transport within the films, resulting in self-induction when a potential difference is applied at high frequencies.

We also carried out EIS for PPA films to gain insight into the charge transport mechanism in those films (Figure S5). Nyquist plots for PPA films demonstrate semicircular behavior which can be fitted with a Randles circuit with a capacitance and a resistive component in parallel (Figure S5a). The magnitude of the impedance follows the same trend as observed in the DC measurements (Figure S5b). Comparing the EIS Nyquist plots for PPA films with PPP films, it is clear that these two kinds of films have different charge transport mechanisms. Since the difference between these two films is the presence of aromatic groups in PPP films and the lack of them in PPA films, it is clear that the introduction of the aromatic sites in the PEDOT:PSS matrix can not only alter the charge transport mechanism but also significantly improve the transport efficiency.

### 2.3 Scanning Microscopy and Conducting Probe AFM (CP-AFM)

**Figure 5:**
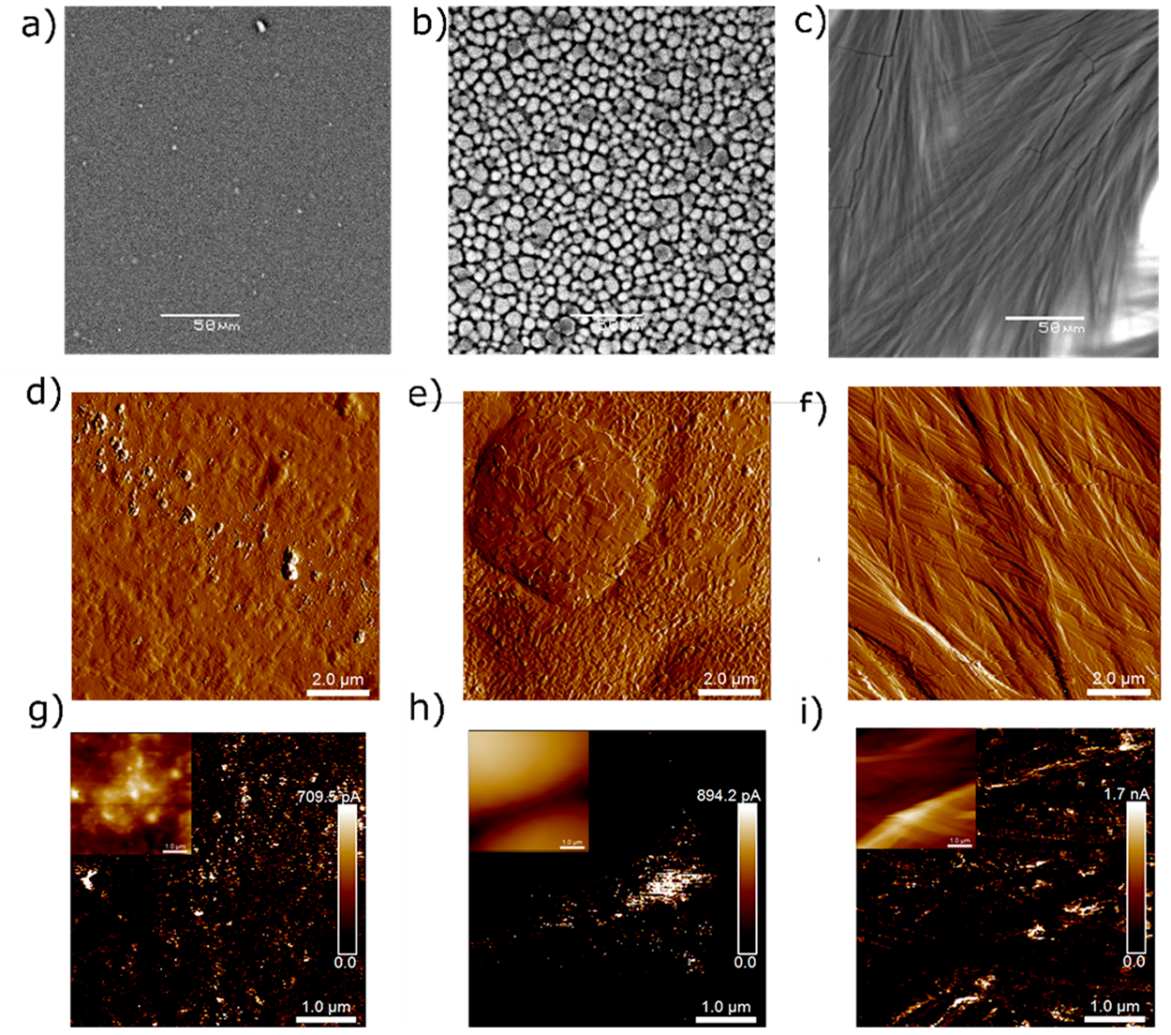
(a–c) SEM images (scale bars: 50 μm), (d–f) AFM topographical images (scale bars: 2 μm), and (g–i) CP-AFM images of PPP films with 0, 50, and 90% Phe, respectively. Scale bars for CP-AFM are 1 μm, and the corresponding topographical AFM images are shown as insets.

In order to investigate if there were any morphological changes in the PPP films as the amount of Phe was increased, we observed the films under SEM and AFM (Figure 5, Figure S6). The surface of a pristine PEDOT:PSS film was found to be essentially flat with minimal surface features (Figure 5a,d). However, when Phe was introduced to create PPP films, we observed that globular structures started appearing, the number of which increased with the increasing concentration of Phe (Figure S6). PPP films with 50 vol % Phe had dense globular structures (Figure 5b) with a diameter in the range of about 5 μm (Figure 5e) distributed throughout the surface. The sizes of globules were smaller toward the center of the circular PPP films and larger at the edges. A closer look at the surface of these globules using AFM demonstrated that the surfaces of these globules were covered with short pieces of self-assembled Phe fibers. When the concentration of Phe was further increased, the globular surface gave way to hierarchical structures (Figure S6), which appeared to be structures formed by the fusion of the elongated self-assembled fibrillar structures with a diminishing number of globules on the surface. The surface of PPP films with 90 vol % Phe was almost entirely composed of the fused structures created by the alignment of multiple Phe fibrils (Figure 5c,f).

We also carried out conducting probe AFM (CP-AFM) on these films to acquire the current map on the film surfaces (Figure 5g–i) so that we can understand which components of the films are responsible for charge transport through PPP films. Pristine PEDOT:PSS films had light current spots distributed throughout the surface (Figure 5g). As expected, the entire surface did not allow the current to pass since the surface of a PEDOT:PSS film has insulating PSS scattered throughout. When we carried out the measurements for PPP films with 50 vol % Phe, we observed that the highly conducting spots were distributed near the edges of the globules (Figure 5h). There were conducting spots on the top of the globule, but those spots were not as conducting as the region around the edge (Figure S9). This makes sense since the globules are covered with fragments of self-assembled Phe fibrils. Nevertheless, it does demonstrate that the materials underneath the filaments on the globules are more conducting than the ones on the surface of pristine PEDOT:PSS, suggesting that the globules are an aggregation of PEDOT units. In the PPP films with 90 vol % Phe, which had their surfaces covered by elongated self-assembled Phe structures, we observed that the conducting spots were prevalent along the edges of the fibril-like structures of Phe. This also suggested that Phe fibrils themselves are not conducting; however, there lies a region rich in PEDOT units underneath that promotes charge transport through the film.

These observations suggest that there are two different mechanisms of self-assembly involved in PPP films depending on the concentration of Phe. The first mechanism involves the appearance and growth in size and number of globular structures on the film surface as the Phe concentration is increased in PPP films. The second mechanism involves the formation of elongated fibrils of Phe fused with neighboring fibrils to create mat-like structures on the surface with a PEDOT-rich conducting region underneath the mat. These observations suggest that while excess Phe in PPP films aggregates into nonconducting fibrils when the film is prepared from the solution phase, the presence of Phe promotes structural changes within PEDOT, resulting in the phase separation of PEDOT:PSS and creating various morphological structures such as globules through which efficient charge transport can occur.

### 2.4 X-ray Diffraction (XRD)

We also carried out XRD measurements on these films (Figure S8) to get further insight into the structure of the PPP films. Despite introducing some Phe into PEDOT:PSS, we do not see any difference in the spectra of PPP films until the concentration of the Phe was higher than the concentration of PEDOT:PSS. This suggests that the globular structures that appear on the surface of the PPP films are not atomically crystalline. The films show crystallinity only after the concentration of the Phe exceeded that of PEDOT:PSS which, based on the SEM images (Figure S6), corresponds to the accumulation of Phe fibrils on the PPP film surface. This suggests that the improvement in charge transport in PPP films is not due to an increase in crystallinity, which further corroborates our hypothesis that the aromatic residues in Phe promote the growth of PEDOT globules in the film which are responsible for providing efficient charge transport pathways due to the removal of PSS.

### 2.5 Stability

Environmental stability^20,38^ of the conducting films is a desired property for their potential application in devices that are exposed to various environmental conditions. PEDOT:PSS has been long known for its environmental stability, which has been one of its attributes that have allowed for a wide use of this polymer.^20,39^ Therefore, as PEDOT:PSS-based composites are developed, there is an interest in studying the stability of those composite materials as well.^40,41^ In order to gain some insight into the long-term stability of PPP films under ambient conditions, we carried out conductivity measurements on those films after a month of their preparation. We observed that the conductivity of the PPP films remains relatively stable (Table S1), suggesting that no significant changes occurred in these films when left out in the room with ambient environmental conditions for a month.

We also investigated whether exposure of the PPP films to water alters conduction through the films. After measuring the conductance of dried PPP films with various vol % Phe, we placed water droplets on the films. We observed that the films demonstrated a slight reduction in conductivity while the films were submerged in the water. However, when the films were again allowed to dry, the film conductance reached the initial levels (Figure S10). This suggests that while the presence of water could slightly impede electron transport, the initial transport mechanism is reinstated after water is removed from the film, suggesting that the films are stable even after exposure to water.

### 2.6 Discussion

Based on the conduction behavior of the PPP films and morphological changes observed in them, we propose that PEDOT:PSS undergoes a phase change with the introduction of Phe. Based on the observations that (1) the conductivity of PPP films increases exponentially with an increasing concentration of Phe from none to 50 vol %, (2) the capacitive component in the charge transport process decreases with an increasing amount of Phe, and (3) conducting globular structures appear on the surface, which increases in size with an increasing concentration of Phe in PPP films, we suggest that the introduction of Phe promotes the growth of PEDOT domains and hence reduces the amount of PSS in between. This then helps create more efficient charge transport paths within the films.

There have been other reports of the formation of globules on the surface of PEDOT:PSS films when they were doped with secondary dopants which enhanced the conductivity of the films. For example, when ethylene glycol (EG) was introduced as a dopant in PEDOT:PSS, globular cluster domains appeared on the surface of the film which was quantified by the surface roughness and the current map which increases in size with an increasing amount of dopant.^11,19,42^ Similar morphological phenomena have also been reported while doping PEDOT:PSS with dimethyl sulfoxide (DMSO).^33,43^ Based on our experimental observation, it appears that similar morphological changes are responsible for enhancing the conduction in PEDOT:PSS films when Phe is introduced. We observed that morphological changes occur in PPA films as well as when the concentration of Ala was increased, but the surface structures are not globular (Figure S7). In the PPA with 50 vol % Ala solution, hierarchical structures appear on the surface due to the aggregation of Ala, but the lack of PEDOT-rich globular structure means that the film does not allow for efficient charge transport as in the case of PPP films.

It is known that the polarity of a dopant also affects the conductivity in PEDOT:PSS films by screening charges and hence limiting the interaction between PEDOT and PSS and inducing phase separation.^13,14^ Such charge screening and phase separation can also be caused by the introduction of zwitterions in the PEDOT:PSS solution.^44^ However, both Ala and Phe have nonpolar residues and are zwitterions with similar isoelectric points of 6 and 5.48, respectively. Therefore, the charge interaction between amino acid and PEDOT:PSS is likely not the reason since the morphological changes occur in PPP and PPA films with different conduction mechanisms. The major difference between these two amino acids is the presence of aromatic amino acid residue in Phe and the lack of it in Ala, which also makes Phe highly hydrophobic. Phe has a relative hydrophobicity of 97 compared to the hydrophobicity of 41 for Ala.^45,46^ It could be that the hydrophobicity due to aromatic residue is responsible for bringing about the morphological changes in the film during the self-assembly process as the PEDOT:PSS–Phe solution mixture dries to form the PPA film. At this point, it is unclear if the aromatic residues in Phe also contribute as sites for charge transport, especially in the PPP films with lower concentrations of Phe. Nevertheless, the fact that an essential amino acid such as Phe can assist with the dramatic enhancement of charge transport through the PEDOT:PSS system takes us one step closer to integrating biological molecules into electronics of the future.

## 3 Conclusions

We have demonstrated that the conductivity of PEDOT:PSS films can be significantly increased by the addition of Phe. At the 50 vol % combination of PEDOT:PSS solution and Phe solution, we were able to enhance the conductivity of the film by about 230 times compared to the conductivity of a pristine PEDOT:PSS film. According to EIS measurements, this increase in conductivity is accompanied by more efficient charge transport through the film with diminishing capacitive components that eventually ceases as the film’s conductivity increases by orders of magnitude with an increasing amount of Phe. SEM and AFM imaging demonstrate that this coincides with the appearance of micrometer-sized globular structures on the film surface which, according to CP-AFM, are conducting clusters potentially rich in PEDOT. Therefore, the efficient charge transport through the film is possibly due to the phase separation of PEDOT from PSS promoted by the prevalence of the aromatic residues. The prospect of using biological materials such as amino acids as dopants to improve the conductivity of PEDOT:PSS, which is used to construct biocompatible electrodes and devices, opens up opportunities in bioelectronics and at the same time helps to realize electronics based on sustainable sources.

## 4 Materials and Methods

### 4.1 Materials

We purchased l-phenylalanine (Phe, 99%) and l-alanine (Ala, 99%) from Fisher Scientific and 1.3% poly(3,4-ethylenedioxythiophene)-poly(styrenesulfonate) (PEDOT:PSS) solution from Millipore Sigma. Thin-film gold interdigitated array microelectrodes (10/10 μm) (ED-IDA1-Au) were purchased from Micrux Technologies.

### 4.2 Preparing Composite Films

We added 0.17 mg of the l-Phe per 1 mL of deionized water in a microcentrifuge tube and vortex mixed with a Fisherbrand digital vortex meter at 3000 rpm for 30 s until most of the solid was dissolved. After letting it settle for 5 min, the Phe solution was mixed with PEDOT:PSS solution to create PEDOT:PSS-Phe (PPP) composites with various volumetric percentages of the constituent solution. The composite solutions ranged from 0 to 100% of the Phe solution with an interval of 10%, with the other portion consisting of PEDOT:PSS. We vortex mixed each of these solutions at 3000 rpm for 60 s and stored them at 5 °C. To create films, we drop-cast 3 μL of the solution onto the interdigitated electrodes. After letting the solution dry for 24 h, this resulted in an approximately circular spot with a mean area of 6.497 ± 0.42 mm^2^, which was calculated using ImageJ. The same procedure was repeated for composite films with Ala.

### 4.3 Conductivity Measurements

The DC electrical measurements were carried out with a Keithley SMU 2612B connected to a probe station (TS150, MPI Corp.) with the help of Keithley Kickstart software to control the SMU. AC electrical measurements were carried out with a Reference 620+ potentiometer (Gamry Instruments) connected to the same probe station. The potentiometer was controlled by Echem Analyst (Gamry) software to set parameters for electrochemical impedance spectroscopy (EIS) measurements. EIS data analysis and modeling were carried out with ZView 4.0 software (Scribner Associates). All electrical measurements were carried out at room temperature and humidity.

The solutions with varying volumetric mixtures were drop-cast on interdigitated electrodes (Micrux Technologies, ED-IDA1-Au) with 10 μm pitch and a 10-μm-wide gold electrode on the glass substrate. For conductivity measurements, the film thickness was measured with P7 Stylus Profilometer (KLA Tencor) or NewView 7300 Optical Profilometer (Zygo) whenever appropriate. The resistance (*R*) values were calculated from the *I*–*V* curves, and the conductivity (σ) values were then calculated using eq 6.

### 4.4 SEM Imaging

A JEOL (JM636OLV) scanning electron microscope (SEM) was used to observe the surface of the composite films. For SEM imaging, we drop-cast 5 μL of the mixture solution onto a silicon dioxide substrate. We left the samples out for 24 h before taking SEM images. The SEM images were taken in BEC mode, with 20 V as the accelerating voltage.

### 4.5 X-ray Spectroscopy

X-ray diffraction (XRD) was carried out on a Philips PW3040 X-ray diffractometer with X’Pert software for extracting data. The instrument uses Cu Kα radiation with a wavelength of 1.54 Å. The 20 mg/mL solution of self-assembled fibrils was drop-cast on glass slides and left to dry overnight. The XRD setup involved a 0.04° soller slit, 1° divergence slit, 1° antiscatter slit, and 1/4° receiving slit.

### 4.6 AFM Studies

AFM imaging of the sample surface was carried out using a Bruker Dimension Icon AFM in ScanAsyst mode with SCANASYST-AIR probes, which are also manufactured by Bruker.

Conducting-probe AFM (CP-AFM) was carried out by using the Bruker Dimension Icon AFM in PF-TUNA mode with PF-TUNA probes coated with Pt/Ir. For the measurements, we prepared the samples by drop-casting 5 μL of the mixture solutions on gold-plated silicon dioxide chips as substrates. A 1 V bias was applied to the substrate while the current mapping was carried out over the surface.

## Supporting Information Available

Optical images of the PPP films, *I*–*V* response of PPA films, EIS Nyquist and Bode plots from PPP films and PPA samples, SEM and AFM images of PPP and PAA films, XRD of PPP films, CP-AFM of PPP film globules, conductance of PPP films before and after exposure to water, and resistance and conductivity values of PPP films (PDF)
