# Supplementary material for: Phenylalanine-Assisted Conductivity Enhancement in PEDOT:PSS Films
Source: ACS Omega. 2023 Feb 15;8(8):7791–9. doi: 10.1021/acsomega.2c07501 (PMC9979372; doi:10.1021/acsomega.2c07501)
Supplement: Supplementary file 1 — ao2c07501_si_001.pdf [file ao2c07501_si_001.pdf]

Phenylalanine Assisted Conductivity Enhancement in PEDOT:PSS Films

*Div Chamria[1], Christopher Alpha[2], Ramesh Y. Adhikari[1]**

[1] Department of Physics & Astronomy, Colgate University, 13 Oak Drive, Hamilton, NY 13346.

[2] Cornell NanoScale Science and Technology Facility, 250 Duffield Hall, Ithaca, NY 14853.

* To whom all correspondence should be addressed: Phone: 315-228-6084 ; E-mail: radhikari@colgate.edu

Table of Contents

Figure S1: Optical images of the dry films of PEDOT:PSS-Phenylalanine (PPP) composite dropcasted onto a substrate with various concentration of phenylalanine a) 0% b) 10% c) 20% d) 30% e) 40% f) 50% g) 60% h) 70% i) 80% j) 90% k) 100%. ……………………………………………………………3

Figure S2: Current-voltage (IV) response of PEDOT:PSS-Alanine (PPA) films. ………………………...4

Figure S3: Nyquist plot of PPP films with the concentration of phenylalanine from a) 0-30% and b) 40-90%. Corresponding equivalent circuits that fits the plot are presented as inset in each of the charts. ……4

Figure S4: Bode plot of PPP films with (a,c) 0-30% phenylalanine and (b,d) 40-90% phenylalanine. …...5

Figure S5: EIS measurements of PPA films with a) Nyquist plot and b) Bode plot. …………………….5

Figure S6: I) SEM and II) AFM images of PPP films with various concentration of phenylalanine a) 0% b) 10% c) 20% d) 30% e) 40% f) 50% g) 60% h) 70% i) 80% j) 90%. …………………………………6

Figure S7: I) SEM and II) AFM images of PPA films with various concentration of alanine a) 0% b) 10% c) 20% d) 30% e) 40% f) 50% g) 60% h) 70% i) 80% j) 90%. …………………………………………6

Figure S8: XRD spectra of PPP films. ………………………………………………………………..7

Figure S9. Current maps on (a) top of a globule and (b) on the edges of globules. ……………………..7

Figure S10. Change in conductance of PPP films after submersion in water. …………………………...8

Table S1: Resistance of the PPP film at a week and a month as well the corresponding conductivity values. ……………………………………………………………………………………………….8

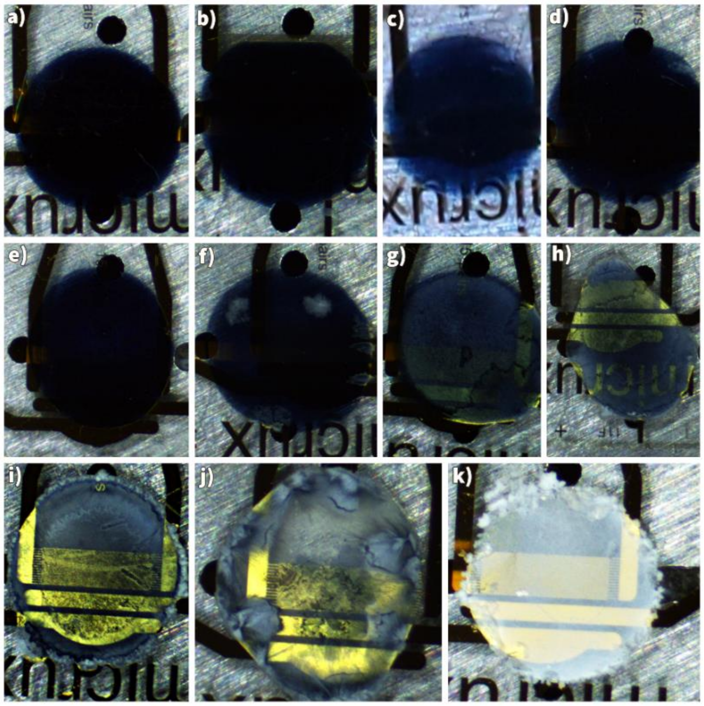

Figure S1: Optical images of the dry films of PEDOT:PSS-Phenylalanine (PPP) composite dropcasted onto a substrate with various concentration of phenylalanine a) 0% b) 10% c) 20% d) 30% e) 40% f) 50% g) 60% h) 70% i) 80% j) 90% k) 100%.

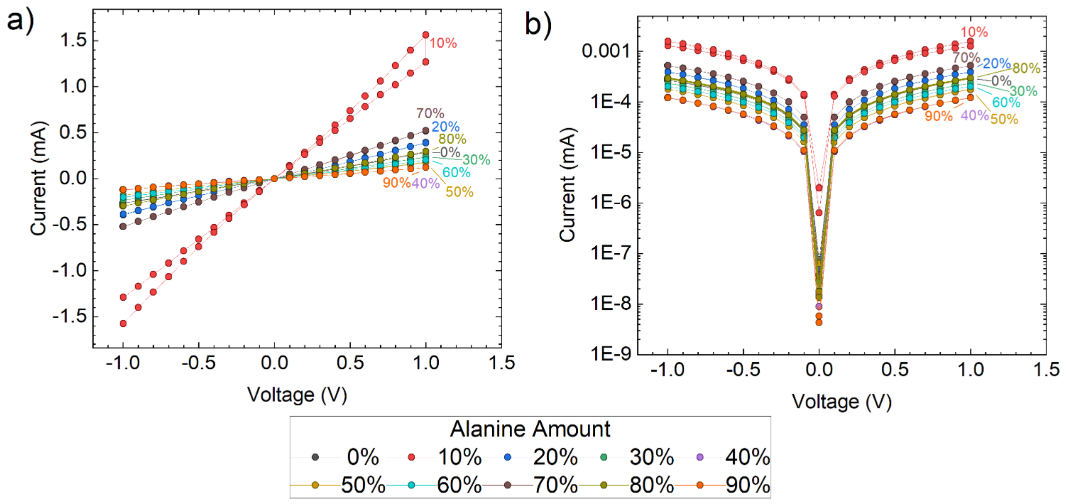

Figure S2: Current-voltage (IV) response of PEDOT:PSS-Alanine (PPA) films.

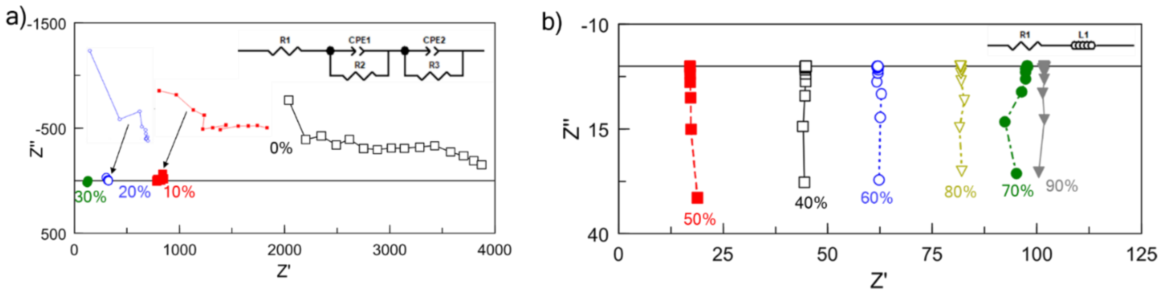

Figure S3: Nyquist plot of PPP films with the concentration of phenylalanine from a) 0-30% and b) 40-90%. Corresponding equivalent circuits that fits the plot are presented as inset in each of the charts.

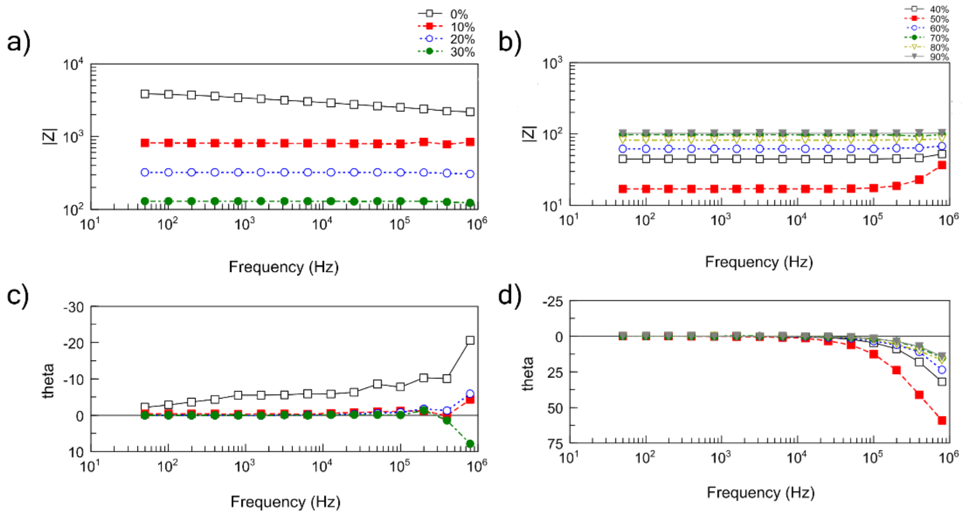

Figure S4: Bode plot of PPP films with (a,c) 0-30% phenylalanine and (b,d) 40-90% phenylalanine.

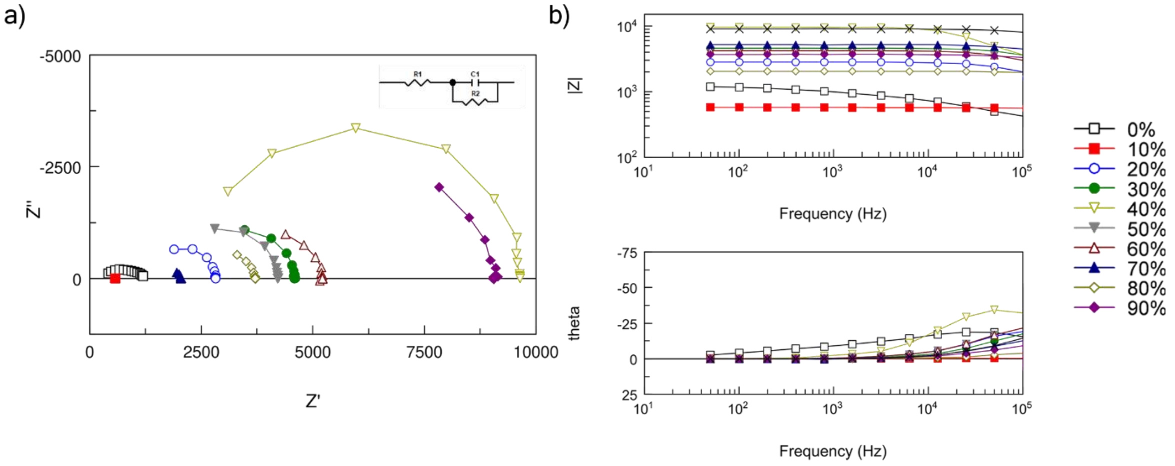

Figure S5: EIS measurements of PPA films with a) Nyquist plot and b) Bode plot.

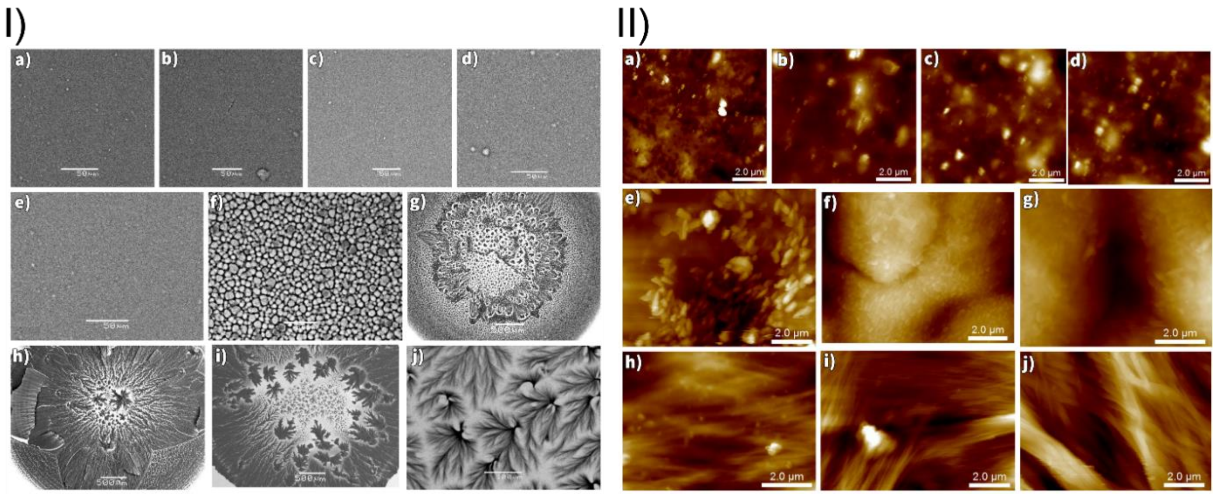

Figure S6: I) SEM and II) AFM images of PPP films with various concentration of phenylalanine a) 0% b) 10% c) 20% d) 30% e) 40% f) 50% g) 60% h) 70% i) 80% j) 90%.

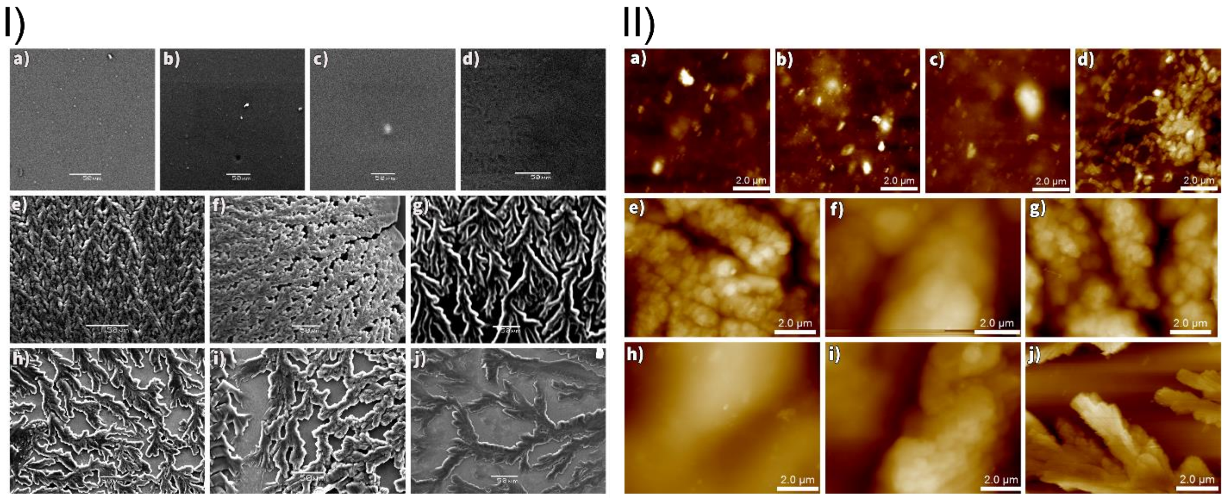

Figure S7: I) SEM and II) AFM images of PPA films with various concentration of alanine a) 0% b) 10% c) 20% d) 30% e) 40% f) 50% g) 60% h) 70% i) 80% j) 90%.

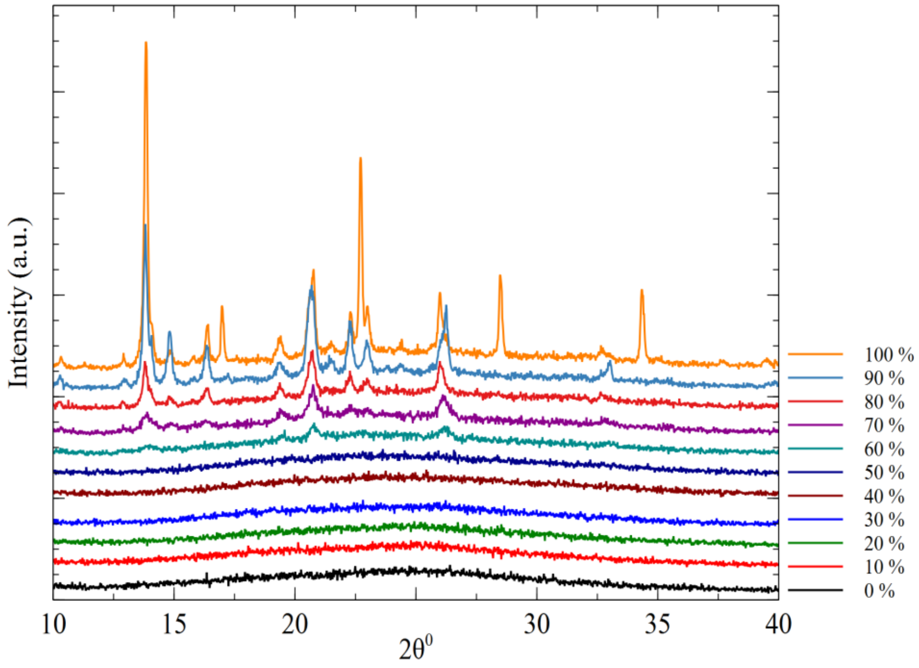

Figure S8: XRD spectra of PPP films.

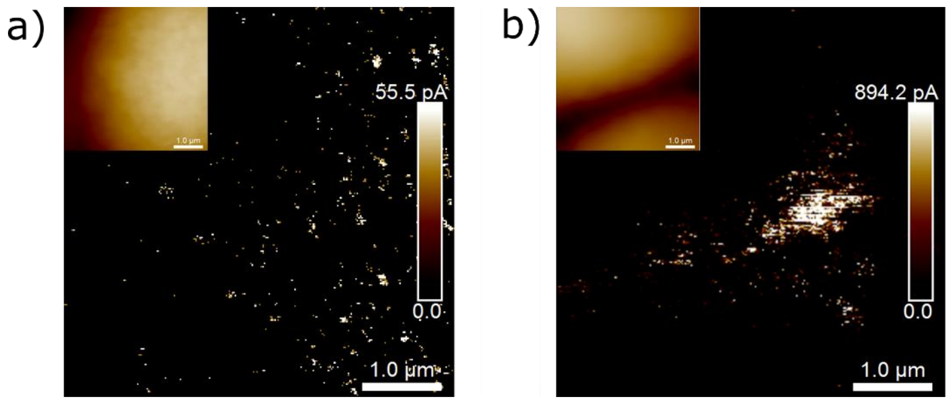

Figure S9. Current maps on (a) top of a globule and (b) on the edges of globules.

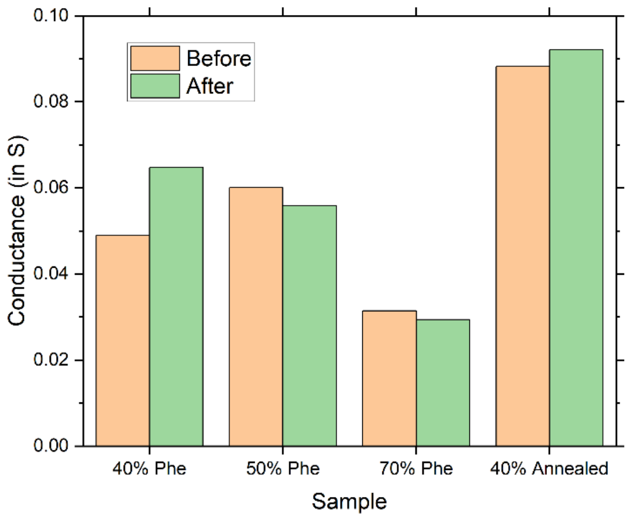

Figure S10. Change in conductance of PPP films after submersion in water.

**Sample** (vol. % of L-Phe in PEDOT:PSS/L-Phe composite)	**DC Resistance (Ω)**		**AC Resistance (Ω)**		**Conductivity (mS/cm)**	
	1 week	1 month	1 week	1 month	1 week	1 month
0	3663 ± 183	2564 ±128	3874 ± 223	2632 ± 170	0.21 ± 0.01	0.29 ± 0.01
10	810 ± 36	654 ± 29	820 ± 10	728 ± 82	0.76 ± 0.03	0.95 ± 0.04
20	293 ± 12	293 ± 12	321 ± 9	321 ± 9	3.06 ± 0.12	3.06 ± 0.15
30	115 ± 5.8	112 ± 5.6	129 ± 15.6	122 ± 11.8	5.14 ± 0.21	5.30 ± 0.26
40	42 ± 1.6	43 ± 1.7	45 ± 3.5	47 ± 4.4	15.00 ± 0.64	14.55 ± 0.71
50	17 ± 0.5	17 ± 0.5	17 ± 0.32	19 ± 1.64	47.33 ± 2.1	46.86 ± 2.3
60	55 ± 2.2	62 ± 2.5	62 ± 7.6	68 ± 6.3	10.53 ± 0.51	9.33 ± 0.42
70	87 ± 3.5	100 ± 3.9	98 ± 12.3	111 ± 12.6	7.73 ± 0.39	6.75 ± 0.34
80	73 ± 1.4	93 ± 1.8	82 ± 9.3	100 ± 7.5	12.79 ± 0.64	10.05 ± 0.5
90	91 ± 5.5	100 ± 6	102 ± 12	110 ± 10.7	11.72 ± 0.84	10.63 ± 0.46

Table S1: Resistance of the PPP film at a week and a month as well the corresponding conductivity values.
